# Mediating Effect of Sleep Disorder Between Low Mental Health Literacy and Depressive Symptoms Among Medical Students: The Roles of Gender and Grade

**DOI:** 10.3389/fpsyt.2022.818295

**Published:** 2022-02-03

**Authors:** Jie Hu, Jun Wang, Danlin Li, Xuexue Huang, Yanni Xue, Liyuan Jia, Zhixian Zhang, Yuhui Wan, Xianbing Song, Rui Wang, Jun Fang, Yehuan Sun, Shichen Zhang

**Affiliations:** ^1^Department of Maternal, Child and Adolescent Health, School of Public Health, Anhui Medical University, Hefei, China; ^2^School of Public Health, Anhui Medical University, Hefei, China; ^3^MOE Key Laboratory of Population Health Across Life Cycle/Anhui Provincial Key Laboratory of Population Health and Aristogenics, Hefei, China; ^4^Department of Human Anatomy, Histology and Embryology, Anhui Medical College, Hefei, China; ^5^Information Technology Office, Anqing Medical College, Anqing, China; ^6^Faculty of Pharmaceutical Science, Sojo University, Kumamoto, Japan; ^7^Department of Epidemiology and Biostatistics, School of Public Health, Anhui Medical University, Hefei, China; ^8^School of Public Health and Health Management, Anhui Medical College, Hefei, China

**Keywords:** mental health literacy, sleep disorder, depressive symptoms, medical students, gender, grade

## Abstract

**Objective:**

In this study, we aimed to disentangle the mediating effect of sleep disorder between mental health literacy (MHL) and depressive symptoms in Chinese medical students, especially focusing on the impact of gender and grade.

**Methods:**

Pooled longitudinal data of 5,504 medical students was collected between November 2019 and June 2020 to assess the MHL, sleep disorder and mental health of medical students in Anhui province, China. Mediation analyses were tested by using bootstrapping procedures.

**Results:**

Sleep disorder were negatively correlated with adequate MHL, but positively correlated with depressive symptoms. The relationships between MHL and depressive symptoms were mediated by sleep disorder in total samples and the indirect effect accounted for 13.59% of the total effect. However, the ratio was 20.82% in female students, whereas no mediating effect was found in the male students. Moreover, the ratio was found higher in freshmen (15.11%) than that in sophomores (11.56%).

**Conclusion:**

Improving the sleep disorder by enhancing MHL is an effective way to reduce depressive symptoms in Chinese medical students. Further investigations elaborately considered by using more gender-balanced population with higher grade and lower level of education.

## Introduction

Depression is a common mental disorder. More than 264 million people of all ages suffer from depression in the world (1). In China, the weighted prevalence of any mental disorder (excluding dementia) was 9.3%, and depressive disorders, anxiety disorders and alcohol use disorders were the common class of disorders (2). Depression is a leading cause of disability worldwide and is a major contributor to the overall global burden of disease (1). Depressive and anxiety disorders will continue to be the major contributors to mental, neurological, and substance use disorder burden, accounting for about 39% of the populations in China (3). In addition, Rotenstein et al. showed that the prevalence of depression or depressive symptoms and suicidal ideation among medical students were 27.2% and 11.1%, respectively, and these symptoms were more common in medical students than other students (4). Many mental disorders relate to poor mental health literacy (MHL). Those youth who have poor MHL levels are more reluctant to seek help, leading to worse health outcome (5). That MHL is related to depression is an important concept and adequate health literacy (HL) is helpful for the prevention, early diagnosis of depression, as well as to improve the prognosis of depression through interventions (6).

MHL refers to knowledge and beliefs about mental disorders which is helpful for the recognition, management or prevention of diseases. MHL has many components, including (a) knowledge of how to prevent mental disorders, (b) recognition of the disease when a disorder is developing, (c) knowledge of help-seeking options and available treatments, (d) knowledge of effective self-help strategies for milder problems, and (e) first aid skills to support others who are developing a mental disorder or are in a mental health crisis (7). Previous studies have shown that improvements in MHL assisted in promoting early detection of mental disorders, and the mental disorders could be halted and even reversed by elevating MHL level, particularly for those individuals who exhibit depression and anxiety (8–10).

A strong link has been elucidated between MHL and depressive symptoms (11), but little is known about the modulating mechanisms or pathway between MHL and depressive symptoms. Understanding the potential pathways modulating the interaction of MHL and depressive symptoms in different populations may thus have a positive impact on intervention strategies. Stewart et al. found that social support (total and subscale scores) mediated the effect of health literacy on depression (12). Previous studies suggested that students who had sleep problems with low HL are at risk of exhibiting anxiety symptoms and depressive symptoms (13). Sleep, as a natural physiological behavior, is closely related to health outcomes (e.g., obesity, cancer, depressive symptoms) (14). The negative aspects of MHL, including discrimination and stigma, may increase one's negative emotions, which is also linked to sleep disorder (15). In contrast, sleep plays an important role in maintaining effective cognitive and interpersonal functions (16). Other positive aspects of MHL, such as effective self-help strategies and help-seeking options may regulate emotional reactivity, which is associated with sleep (17). Namely, emotional support relations are associated with better sleep quality.

A growing number of studies have further indicated the relation between sleep disorder and depressive symptoms (14, 18). A cohort study demonstrated that sleep deprivation also increased the risk of depressive symptoms in which a reciprocal effect existed (19). Moreover, a meta-analysis of randomized controlled trials showed that non-pharmacological sleep interventions could improve the severity of depression symptom efficiently (20). Despite methodological shortcomings, these studies clearly implied that depression could be improved in the context of sleep quality by using intervention strategies. Taken together, a strong link exists between low MHL and depressive symptoms in which sleep disorder may play a critical role for mediating this relation. However, so far, most studies regarding this issue tend to treat the respondents as a homogeneous group, but the group differences are less considered.

Previous studies found the distinct distribution pattern of MHL, sleep disorder and depressive symptoms in male and female, which suggested gender may potentially impact the mediating effect of sleep disorder between low MHL and depressive symptoms (21, 22). Meanwhile, the grade of students (i.e., the age) was found to a key factor associated with MHL, sleep disorder and depressive symptoms (23–25). In this context, it is important to investigate the potential impact of demographic characteristics of the population, especially gender and grade differences, in the interaction of MHL and mental disorders, which may have important implications for school intervention policies. For this aim, in this study we identified the associations between low MHL, sleep disorder, and depressive symptoms, especially focusing on the impact of gender and grade on the mediating effect of sleep disorder in the associations between low MHL and depressive symptoms.

## Methods

### Design and Participants

**Figure 1:**
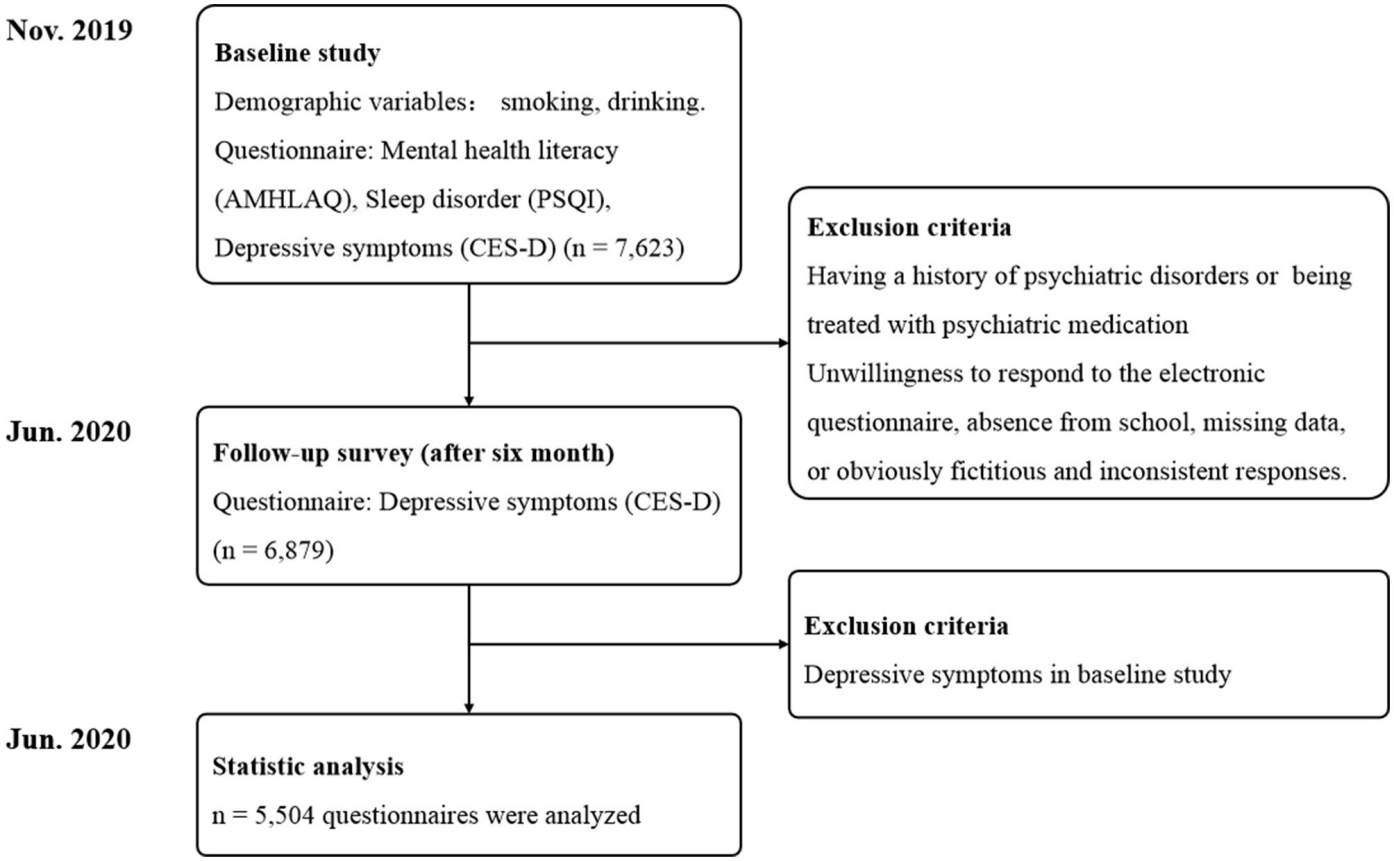
Flow chart of the study.

This longitudinal study was performed between November 2019 and June 2020 to assess the MHL, sleep quality and depressive symptoms in medical students. Participants were recruited from two medical colleges by a cluster sampling method in Anhui province, China. The participants of this study consisted of freshmen and sophomores, and excluding participants who have a history of psychiatric disorders or are being treated with psychiatric medication. We first carried out the baseline survey in November 2019 and the follow-up surveys were completed in June 2020 (6-month follow up) (Figure 1). Written informed consents were obtained from all participants prior to the implementation of the investigation. A link was given to the students, allowing them to access the electronic questionnaire. The students were asked to complete an anonymous questionnaire. Completion of the questionnaire took approximately 20–30 min. A research staff was responsible for the quality control of the questionnaire to answer the questions from the recipients and to proofread the questionnaire.

Data on demographic information, MHL and sleep disorder were collected through electronic questionnaire in the baseline study. A total 7,623 adolescents (1,971 males, 5,652 females) aged 19.66 years (SD = 1.11) who had complete baseline data of MHL and sleep disorder, were selected for the follow-up study, which mainly focused on the mental health of the participants (Figure 1). Among those students qualified the follow-up study, 744 students were excluded from the study because of an unwillingness to respond to the electronic questionnaire, absence from school, missing data, or obviously fictitious and inconsistent responses. Accordingly, the data from 6,879 (90.24%) participants were collected in follow-up study. Given that follow-up depressive symptoms may be the continuance of the symptoms at the time of baseline study, we only analyzed participants without depressive symptoms in baseline study. Finally a total of 5,504 students were selected in this study, with a mean age of 19.65 ± 1.11 years, among which there were 1,347 male students, 4,157 female students, 3,046 freshmen and 2,458 sophomores (Figure 1). The demographic characteristic of participants was presented in (Table 1).

**Table 1:** The demographic characteristic of participants.

**Variable**	**Total sample (%)**
**Gender**	
Male	1,347 (24.47)
Female	4,157 (75.53)
**Grade**	
Freshman	3,046 (55.34)
Sophomore	2,458 (44.66)
**Registered residence**	
Rural	3,533 (64.19)
Urban	1,971 (35.81)
**Any siblings**	
Yes	4,354 (79.11)
No	1,150 (20.89)
**Father's educational level**	
No father	116 (2.11)
< High school degree	4,052(73.62)
≥High school degree	1,336 (24.27)
**Mother's educational level**	
No mother	66 (1.20)
< High school degree	4,584 (83.28)
≥High school degree	854 (15.52)
**Self-reported family economy**	
Bad	1,878 (34.12)
Normal	3,445 (62.59)
Good	181 (3.29)

### Variables

#### MHL

The measurement of MHL was based on the Adolescent Mental Health Literacy Assessment Questionnaire (AMHLAQ), which consists of 22 questions in 4 dimensions, as follows: (1) 6 items regarding knowledge (e.g., “The duration of symptoms of mental problems is an important factor in the diagnosis of mental illness.”); (2) 5 items regarding recognition (e.g., “People with social phobia usually become extremely nervous and anxious when communicating with others or participating in group activities.”); (3) 6 items regarding attitude (e.g., “I think people with mental illness usually come from low-income families.”); and (4) 5 items of practice of (e.g., “If my relatives or friends had mental illness, I would listen to her/him without judging or criticism.”) (26). Participants selected an answer by using 5-point Likert responses (strongly disagree, disagree, neither agree nor disagree, agree, and strongly agree). The scores for all items ranged between 22 and 110. A higher total score indicates a higher level of MHL. In this study, the Cronbach's α coefficient for AMHLAQ was 0.841, and Cronbach's α of each dimensions was 0.681–0.805.

#### Sleep Disorder

Sleep disorder were evaluated by the Pittsburgh Sleep Quality Index (PSQI), which is a self-reported questionnaire containing 7 domains, i.e., sleep quality, habitual sleep efficiency, sleep latency, sleep disturbances, medication use, sleep duration, and diurnal dysfunctions, over the past month (27). The total of scores range 0–21, where higher scores regard poorer sleep quality. Previous studies using PSQI indicated the strong reliability and validity, and moderate structural validity of PSQI in a variety of samples, with the Cronbach's α coefficient ranged from 0.70 to 0.83, suggesting the utility of this tool (28). The Cronbach's α coefficient for PSQI was found 0.744 in this study.

#### Depressive Symptoms

The Center for Epidemiologic Studies Depression Scale (CES-D) compiled by Radloff was used to evaluate depressive symptoms by measuring the frequency of events and ideas (29). CES-D is a 20-item instrument with 4 options ranging from 0 (“rarely or none of the time”) to 3 (“most or all of the time”). The total score ranges from 0 to 60 and a higher score indicates a greater risk of depressive symptoms. Previous study suggested a good reliability and validity of CES-D for assessing subthreshold depression in Chinese university students (30). In this study, the Cronbach's α coefficient for the scale was 0.864.

#### Covariates

The potential correlations of depressive symptoms with demographic characteristics including age, gender, grade, registered residence, any siblings, parental educational level, self-reported family economy, smoking and drinking, were also investigated in this study.

### Analyses

All data were performed using SPSS (version 23.0, SPSS Inc., Chicago, IL, USA). First, descriptive analysis was performed to explore the possible of gender and grade differences. Given the gender and grade difference in description, we conducted a stratified analysis between male and female, as well as between freshman and sophomore. Next, we performed spearman correlation analysis to test the associations among MHL, sleep disorder and depressive symptoms. Then, the mediation pathway from MHL to depressive symptoms through sleep disorder was tested as recommended by Hayes, using bootstrapping procedures with 5,000 random samplings (31). The index of the indirect effect is regarded statistically significant if the 95% confidence interval does not contain 0. Finally, we examined whether sleep disorder mediated the relationship between low MHL and depressive symptoms in different gender and grade.

## Results

### Correlations Among of MHL, Sleep Disorder and Depressive Symptoms Among Chinese Medical Students

Gender and grade differences in descriptive statistics related to different dimensions of MHL, sleep disorder and depressive symptoms were presented in (Table 2). Correlation analysis between key variables were presented in (Table 3). Although we used stratified analysis in this study, we also reported the results of the correlation analysis of the total samples. Spearman correlation analysis among these key variables were in expected directions (e.g., sleep disorder was negatively correlated with adequate MHL, but positively correlated with depressive symptoms). The exceptions were that in male no significant correlations were found for knowledge, attitude and recognition with sleep disorder. Also, in female no significant correlations were found for recognition with depressive symptoms. In addition, with regard to recognition, between freshmen and sophomore participants, no statistically significant differences were found for depressive symptoms and sleep disorder (*p* > 0.05, Table 3).

**Table 2:** Descriptive statistics for variables.

**Variable**	**Total** **(*n* = 5,504)** **Mean ±SD/** ***M* (*P_25_*, *P_75_*)**	**Sex**		**Grade**	
		**Male** **(*n* = 1,347)**	**Female** **(*n* = 4,157)**	**Freshman** **(*n* = 3,046)**	**Sophomore** **(*n* = 2,458)**
		**Mean ±SD/** ***M* (*P_25_*, *P_75_*)**	**Mean ±SD/** ***M* (*P_25_*, *P_75_*)**	**Mean ±SD/** ***M* (*P_25_*, *P_75_*)**	**Mean ±SD/** ***M* (*P_25_*, *P_75_*)**
Mental health literacy	85.96 ± 6.97	85.72 ± 8.26	86.04 ± 6.50	85.82 ± 6.80	86.14 ± 7.18
Knowledge	24.08 ± 2.57	24.16 ± 2.95	24.05 ± 2.43	23.90 ± 2.57	24.30 ± 2.55
Recognize	19.62 ± 2.41	19.48 ± 2.79	19.67 ±2.27	19.55 ± 2.43	19.70 ± 2.37
Attitude	22.25 ± 2.35	21.93 ± 2.58	22.35 ± 2.27	22.37 ± 2.28	22.09 ± 2.43
Practice	20.02 ± 2.17	20.16 ± 2.45	19.97 ± 2.06	19.99 ± 2.13	20.05 ± 2.22
Sleep Disorder	4.00 (3.00, 6.00)	4.00 (3.00, 6.00)	4.00 (3.00, 6.00)	5.00 (3.00, 6.00)	4.00 (2.00, 6.00)
Depressive symptoms	7.00 (3.00, 12.00)	8.00 (4.00, 12.00)	7.00 (3.00, 12.00)	8.00 (4.00, 12.00)	7.00 (3.00, 12.00)

*SD, standard deviation; M, median; P_25_, lower quartile; P_75_, upper quartile; The normally distributed data is described as Mean ± SD; The non-normally distributed data is described as M (P_25_, P_75_)*.

**Table 3:** Correlations of MHL, sleep disorder and depressive symptoms among Chinese medical students.

**Variable**		**1**	**2**	**3**	**4**	**5**	**6**	**7**
**Gender**								
Male	1.MHL	1						
	2.Knowledge	0.811^**^	1					
	3.Recognition	0.744^**^	0.659^**^	1				
	4.Attitude	0.568^**^	0.233^**^	0.141^**^	1			
	5.Practice	0.771^**^	0.602^**^	0.482^**^	0.304^**^	1		
	6.Sleep disorder	−0.070^**^	−0.048	−0.026	−0.033	−0.079^**^	1	
	7.Depressive symptoms	−0.199^**^	−0.110^**^	−0.085^**^	−0.221^**^	−0.192^**^	0.188^**^	1
Female	1.MHL	1						
	2.Knowledge	0.757^**^	1					
	3.Recognition	0.673^**^	0.550^**^	1				
	4.Attitude	0.583^**^	0.191^**^	0.135^**^	1			
	5.Practice	0.678^**^	0.448^**^	0.320^**^	0.253^**^	1		
	6.Sleep disorder	−0.115^**^	−0.103^**^	−0.038^**^	−0.047^**^	−0.151^**^	1	
	7.Depressive symptoms	−0.144^**^	−0.067^**^	−0.018	−0.157^**^	−0.164^**^	0.212^**^	1
**Grade**								
Freshman	1.MHL	1						
	2.Knowledge	0.770^**^	1					
	3.Recognition	0.688^**^	0.552^**^	1				
	4.Attitude	0.572^**^	0.200^**^	0.132^**^	1			
	5.Practice	0.691^**^	0.454^**^	0.325^**^	0.281^**^	1		
	6.Sleep disorder	−0.121^**^	−0.103^**^	−0.052^**^	−0.055^**^	−0.139^**^	1	
	7.Depressive symptoms	−0.152^**^	−0.071^**^	−0.025	−0.175^**^	−0.175^**^	0.197^**^	1
Sophomore	1.MHL	1						
	2.Knowledge	0.775^**^	1					
	3.Recognition	0.697^**^	0.612^**^	1				
	4.Attitude	0.592^**^	0.213^**^	0.149^**^	1			
	5.Practice	0.715^**^	0.539^**^	0.408^**^	0.245^**^	1		
	6.Sleep disorder	−0.079^**^	−0.050^*^	−0.005	−0.044^*^	−0.127^**^	1	
	7.Depressive symptoms	−0.167^**^	−0.071^**^	−0.050^*^	−0.190^**^	−0.160^**^	0.204^**^	1
Total	1.MHL	1						
	2.Knowledge	0.770^**^	1					
	3.Recognition	0.692^**^	0.577^**^	1				
	4.Attitude	0.578^**^	0.200^**^	0.138^**^	1			
	5.Practice	0.702^**^	0.489^**^	0.362^**^	0.263^**^	1		
	6.Sleep disorder	−0.103^**^	−0.089^**^	−0.035^*^	−0.042^**^	−0.134^**^	1	
	7.Depressive symptoms	−0.158^**^	−0.076^**^	−0.037^**^	−0.176^**^	−0.168^**^	0.206^**^	1

* *p < 0.05;*

** *p < 0.01; MHL is mental health literacy*.

### Mediation Effects of Sleep Disorder in the Relationship Between MHL and Depressive Symptoms

To investigate possible role of sleep disorder in the mediation pathway of depressive symptoms, a series of mediating analyses were carried out by using a stratified analysis. Mediation models were only tested if the 95% CI do not include 0 in all three pathways (a, b, c) (32). The relationship between MHL and depressive symptoms was mediated by sleep disorder in total samples; in the model 4, the indirect effect accounted for 13.59% of the total effect in the total samples (Table 4). However, the ratio of MHL on depressive symptoms was inconsistent according in different genders and grades. Results showed that sleep disorder did not mediate the relationship between MHL and depressive symptoms in male samples, whereas the mediation ratio was 20.82% in the model 2 in female samples. In the model 3, the indirect effect accounted for 15.11% of the total effect among freshmen, while the ratio among sophomores was 11.56% (Table 4). The relationship between different dimensions of MHL and depressive symptoms was not mediated by sleep disorder in males, except for practice dimension. In addition to recognition dimension, sleep disorder plays a mediating role between cognition, attitude and practice, and depressive symptoms among freshmen and sophomores. Sleep disorder mediated the relationships between all dimensions of MHL and depressive symptoms in females (refer to attached Tables A1–A4).

**Table 4:** Mediating effect of sleep disorder between MHL and depressive symptoms.

**Variable**	**Model**	**a**	**b**	**Direct effect**	**Boot CI**		**Indirect effect boot CI**			**Mediation ratio, %**
				**c'**	**LLCI**	**ULCI**	**(a × b)**	**LLCI**	**ULCI**	**(a × b)/(a × b + c')**
**Gender**										
Male	1	−0.0162	0.3729^**^	−0.1354^**^	−0.1746	−0.0963	−0.006	−0.0139	0.0007	-
	2	−0.0134	0.3577^***^	−0.1303^***^	−0.1694	−0.0912	−0.0048	−0.0123	0.0015	-
Female	1	−0.0483^***^	0.5401^**^	−0.096^**^	−0.1234	−0.0687	−0.0261	−0.0338	−0.0188	21.38
	2	−0.0476^***^	0.5242^***^	−0.0952^**^	−0.1226	−0.0678	−0.0250	−0.0327	−0.0178	20.82
**Grade**										
Freshman	1	−0.0406^***^	0.487^**^	−0.1153^***^	−0.1473	−0.0833	−0.0198	−0.0285	−0.0124	14.67
	3	−0.0411^***^	0.4836^***^	−0.1118^***^	−0.1439	−0.0797	−0.0199	−0.0286	−0.0123	15.11
Sophomore	1	−0.0312^**^	0.4681^***^	−0.1055^***^	−0.1362	−0.0747	−0.0146	−0.0225	−0.0076	12.17
	3	−0.0289^***^	0.4673^***^	−0.1032^***^	−0.1340	−0.0724	−0.0135	−0.0215	−0.0067	11.56
Total	1	−0.0371^***^	0.4919^***^	−0.1111^***^	−0.1334	−0.0888	−0.0183	−0.0242	−0.0128	14.14
	4	−0.0357^***^	0.4762^***^	−0.1081^***^	−0.1304	−0.0858	−0.0170	−0.0227	−0.0119	13.59

** *p < 0.01;*

*** *p < 0.001;*

*a: Effect of MHL on sleep disorder; b: Effect of sleep disorder on depressive symptoms.*

*MHL is mental health literacy.*

*Model 1: Single factor analysis.*

*Model 2: Adjusted for age, grade, registered residence, any siblings, parents' education level, self-reported family economy, smoking, drinking.*

*Model 3: Adjusted for age, gender, registered residence, any siblings, parents' education level, self-reported family economy, smoking, drinking.*

*Model 4: Adjusted for age, gender, grade, registered residence, any siblings, parents' education level, self-reported family economy, smoking, drinking*.

## Discussion

In this study we explored the association between MHL and depressive symptoms among Chinese medical students. Moreover, our study adds to the literature by emphasizing that sleep disorder is the mediator between MHL and depressive symptoms and this mediating effect is varied by gender and grade. Our study found that adequate MHL was related negatively with sleep disorder and depressive symptoms. Though previous study has already indicated that low HL was related with sleep disorder and depressive symptoms; the evaluation tool of MHL was not used directly (13). The current study is not only a support of previous results but also an extension of the research. It has been commonly understood that sleep disorder often precede the onset of depression and constitute an independent risk factor for depression (33). In line with those findings, our present study indicated that poor sleep quality was positively associated with depressive symptoms.

Stewart et al. indicated that inadequate HL had an indirect impact on depression through its association with social support (12). Moreover, previous studies showed that sleep disorder, as a mediating mechanism, linked psychosocial stressors (e.g., self-blame, self-shame, self-stigma, discrimination) to mental disorder (15, 34). Individuals who were characterized by more shame, self-stigma, less perceived knowledge, higher satisfaction with their mental health that may be barriers to help-seeking, usually companied with low MHL (35). Also, such negative thoughts could adversely affect sleep quality, contributing to mental disorders (15). In accordance with previous psychological studies, our results concluded that inadequate MHL showed an indirect relationship with depressive symptoms that is likely to be achieved by sleep disorder. Interestingly, in male students, the effect of MHL on depressive symptoms seemed not to be affected by sleep disorder in adjusted models. There is evidence that, the masculinity has been regarded as association with low MHL and mental health help-seeking and males may be inclined to conceal their symptoms and experience of depression compared to females so that their behavior conforms to social conventions (36). Masculinity emphasis on competition, strength, avoiding emotions and perceived femininity, an action-orientation, and the acceptability of anger and violence (37). Males are at risk of psychological stress when they are unlikely to seek help for their problems. Namely, males may suppress their emotional expression, or may be ill-equipped to recognize signs of negative emotion and to respond due to low MHL. This may also be the reason why males are less affected by sleep disorder compared with females. On the contrary, the important finding in this study was that low MHL might lead to decreased sleep quality, thus contributing to depressive symptoms in females. Further studies are warranted to confirm this notion which is of importance for the design of the intervention program for university students to improve their mental health.

When covariates were adjusted, the ratio of total effect of MHL on depressive symptoms that was explained by mediation *via* sleep disorder and individuals with freshmen was higher than sophomores. It is not surprising that attending college for the first time can be a challenging experience for freshmen. Studies on transition theory have shown that a passage from one fairly stable state to another fairly stable state, is a process triggered by changes (38). There are many risk factors, such as academic achievement, financial situation, socializing and living environment that affect college student' health (39). To adapt to the new environment, individuals have to deal with the problems, challenges and needs in daily life (40). Previous study has shown that across the transitional first year of college, freshmen experience significantly declines in psychological wellbeing and cognitive-affective strengths as well as remarkable increases in psychological distress and cognitive-affective vulnerabilities (41). Furthermore, Evans et al. indicated that students in upper classes had higher HL, namely, with the increase of academic level, the level of HL is also improving (42). Therefore, freshmen may not be able to solve their predicaments due to inadequate MHL, and they are prone to negative coping styles. Once those with a negative tendency of coping style showed signs of sleep disorder, the possibility of mental problems increased significantly (43).

Medicine is a challenging major with a very stressful environment, and the students are usually required to participate in a wide range of courses, numerous academic requirements and various types of examination (44). The reactions of individuals to stress are quite different, if it is not recognized or managed correctly and timely, stress may manifest in unfavorable ways, such as impaired sleep quality and depressive symptoms (43). In light of the present results, interventions targeting those with low MHL should include improving sleep quality to promote mental health. More specifically, gender and grade differences should also be considered for targeted MHL approaches. Once individuals have sleep disorder, females and freshmen with low MHL were more likely to have depressive symptoms. In this context, intervention to enhance MHL of medical students should be performed as soon as possible to prevent the potential knock-on effects on health, especially among females. Moreover, it should also be noted that though sleep disorder is an important issue, intervention for MHL but not only for sleep disorder, may be more effective in improving health because MHL constitute an independent risk factor for sleep disorder and depressive symptoms.

## Strengths and Limitations

The main contribution of this study is to demonstrate what extent MHL is associated with depressive symptoms through sleep disorder. To our knowledge, our study is the first to examine whether the gender and grade moderate the mediating effects of sleep disorder between low MHL and depressive symptoms in Chinese medical students, which may help to understand the potential negative effects of low MHL and sleep disorder, and to design targeted intervention programs to promote mental health. In addition, strengths of the study also include its longitudinal design, large sample size, and a low level of missing information among participants. However, some limitations exist in this study. Firstly, electronic questionnaires were collected through self-report measure which may be subject to reporting bias. Secondly, although the study samples are enough for difference analysis, and the response rate is relatively high (i.e., 90.24%), the generalizability of the findings is limited as a result because all participants were from two medical colleges. Thirdly, only freshmen and sophomores were included in the sample, and they were all in lower grades and could not reflect all medical students. Finally, although a series of covariates were adjusted in the analyses, some variables that are known to have associations with depressive symptoms, such as family medical history, have not been measured.

## Conclusion

In this study, we demonstrate that sleep disorder is not only a consequence of low MHL, but also a risk factor for depressive symptoms, and it mediates the association between low MHL and depressive symptoms, which the mediating effect is more pronounced in females and freshmen. Our findings suggest that improvement of the sleep disorder of Chinese medical students by enhancing MHL is an effective way to reduce depressive symptoms. Further study using a sample with a more gender-balanced, more types of schools, and different levels of education, is warranted to support and confirm this notion.

## Data Availability Statement

The original contributions presented in the study are included in the article/Supplementary Material, further inquiries can be directed to the corresponding authors.

## Ethics Statement

The studies involving human participants were reviewed and approved by Ethics Committee of Anhui Medical University (approval number 20170290). Written informed consent to participate in this study was provided by the participants' legal guardian/next of kin.

## Author Contributions

JH, JW, XS, and RW were involved in data collection. JH, JW, DL, XH, YX, LJ, and ZZ conducted the Statistical analysis. JH and JW wrote the first draft of the paper, which was critically revised by SZ, YS, and JF. SZ, JW, and JF provided funding for the project. The final manuscript was approved by all authors and contributed to interpretation of the findings.

## Funding

This research was funded by National Ministry of Education Humanities and Social Science Research Planning Fund Project (21YJAZH120), National Natural Science Foundation of China (81402699 and 81573512) and the Natural Science Foundation in Higher Education of Anhui (KJ2020A0209 and KJ2020A0208). The funder had no role in study design, data collection and analysis, decision to publish, or preparation of the manuscript.

## Conflict of Interest

The authors declare that the research was conducted in the absence of any commercial or financial relationships that could be construed as a potential conflict of interest.

## Publisher's Note

All claims expressed in this article are solely those of the authors and do not necessarily represent those of their affiliated organizations, or those of the publisher, the editors and the reviewers. Any product that may be evaluated in this article, or claim that may be made by its manufacturer, is not guaranteed or endorsed by the publisher.
