# Supplementary material for: Mediating Effect of Sleep Disorder Between Low Mental Health Literacy and Depressive Symptoms Among Medical Students: The Roles of Gender and Grade
Source: Front Psychiatry. 2022 Feb 3;13:818295. doi: 10.3389/fpsyt.2022.818295 (PMC8855242; doi:10.3389/fpsyt.2022.818295)
Supplement: Supplementary file 1 [file Data_Sheet_1.docx]

Table 1A Mediating effect of sleep quality between knowledge and depressive symptoms

| Variable | Model | a | b | Direct Effect | Boot CI | |  | Indirect Effect | Boot CI | |  | Mediation Ratio, % |
| --- | --- | --- | --- | --- | --- | --- | --- | --- | --- | --- | --- | --- |
|  |  |  |  | c’ | LLCI | ULCI |  | (a×b) | LLCI | ULCI |  | (a×b)/(a×b+c’) |
| Gender |  |  |  |  |  |  |  |  |  |  |  |  |
| Male | 1 | -0.0356 | 0.3836^***^ | -0.2368^**^ | -0.3476 | -0.126 |  | -0.0137 | -0.0368 | 0.0057 |  | - |
|  | 2 | -0.0241 | 0.3683^***^ | -0.2208^**^ | -0.3316 | -0.1099 |  | -0.0089 | -0.0294 | 0.0088 |  | - |
| Female | 1 | -0.1086^**^ | 0.5634^**^ | -0.0709 | -0.1441 | 0.0024 |  | -0.0612 | -0.0821 | -0.0418 |  | - |
|  | 2 | -0.0990^***^ | 0.5426^***^ | -0.0603 | -0.1338 | 0.0132 |  | -0.0537 | -0.0733 | -0.0360 |  | - |
| Grade |  |  |  |  |  |  |  |  |  |  |  |  |
| Freshman | 1 | -0.0865^***^ | 0.5104^***^ | -0.1197^**^ | -0.2048 | -0.0347 |  | -0.0442 | -0.0665 | -0.0244 |  | 26.97 |
|  | 3 | -0.0870^***^ | 0.5067^***^ | -0.1158^**^ | -0.2010 | -0.0307 |  | -0.0441 | -0.0658 | -0.0244 |  | 27.58 |
| Sophomore | 1 | -0.0641^**^ | 0.4860^***^ | -0.1146^*^ | -0.2019 | -0.0274 |  | -0.0312 | -0.0524 | -0.0116 |  | 21.40 |
|  | 3 | -0.0581^**^ | 0.4840^***^ | -0.1105^*^ | -0.1979 | -0.0232 |  | -0.0281 | -0.0501 | -0.0082 |  | 21.27 |
| Total |  |  |  |  |  |  |  |  |  |  |  |  |
|  | 1 | -0.0855^***^ | 0.5116^***^ | -0.1248^***^ | -0.1858 | -0.0638 |  | -0.0437 | -0.0591 | -0.0291 |  | 25.93 |
|  | 4 | -0.0749^***^ | 0.4964^***^ | -0.1147^***^ | -0.1758 | -0.0536 |  | -0.0372 | -0.0518 | -0.0233 |  | 24.49 |

^*^*p <* 0.05; ^**^*p <* 0.01; ^***^*p <* 0.001;

a: Effect of knowledge on sleep quality; b: Effect of sleep quality on depressive symptoms;

Model 1: Single factor analysis;

Model 2: Adjusted for age, grade, registered residence, any siblings, parents' education level, self-reported family economy, smoking, drinking;

Model 3: Adjusted for age, gender, registered residence, any siblings, parents' education level, self-reported family economy, smoking, drinking;

Model 4: Adjusted for age, gender, grade, registered residence, any siblings, parents' education level, self-reported family economy, smoking, drinking;

Table 2A Mediating effect of sleep quality between recognition and depressive symptoms

| Variable | Model | a | b | Direct Effect | Boot CI | |  | Indirect Effect | Boot CI | |  | Mediation Ratio, % |
| --- | --- | --- | --- | --- | --- | --- | --- | --- | --- | --- | --- | --- |
|  |  |  |  | c’ | LLCI | ULCI |  | (a×b) | LLCI | ULCI |  | (a×b)/(a×b+c’) |
| Gender |  |  |  |  |  |  |  |  |  |  |  |  |
| Male | 1 | -0.0093 | 0.3917^***^ | -0.2106^**^ | -0.3276 | -0.0937 |  | -0.0037 | -0.0249 | 0.0168 |  | - |
|  | 2 | -0.0008 | 0.3748^***^ | -0.1942^**^ | -0.3110 | -0.0775 |  | -0.0003 | -0.0198 | 0.0195 |  | - |
| Female | 1 | -0.0624^***^ | 0.5693^**^ | -0.0217 | -0.1001 | 0.0567 |  | -0.0355 | -0.0570 | -0.0155 |  | - |
|  | 2 | -0.0597^***^ | 0.5473^**^ | -0.0179 | -0.0962 | 0.0605 |  | -0.0330 | -0.0537 | -0.0136 |  | - |
| Grade |  |  |  |  |  |  |  |  |  |  |  |  |
| Freshman | 1 | -0.0478^**^ | 0.5183^***^ | -0.0661 | -0.1558 | 0.0236 |  | -0.0248 | -0.0463 | -0.0039 |  | - |
|  | 3 | -0.0491^**^ | 0.5146^***^ | -0.0576 | -0.1474 | 0.0322 |  | -0.0253 | -0.0479 | -0.0044 |  | - |
| Sophomore | 1 | -0.0301 | 0.4902^***^ | -0.1117^*^ | -0.2053 | -0.0182 |  | -0.0148 | -0.0363 | 0.0059 |  | - |
|  | 3 | -0.0268 | 0.4879^***^ | -0.1032^*^ | -0.1969 | -0.0095 |  | -0.0131 | -0.0352 | 0.0076 |  | - |
| Total |  |  |  |  |  |  |  |  |  |  |  |  |
|  | 1 | -0.0442^**^ | 0.5187^***^ | -0.0892^*^ | 0.0071 | -0.1542 |  | -0.0229 | -0.0386 | -0.0076 |  | 20.41 |
|  | 4 | -0.0405^**^ | 0.5022^***^ | -0.0787^*^ | -0.1436 | -0.0138 |  | -0.0203 | -0.0358 | -0.0058 |  | 20.48 |

^*^*p <* 0.05; ^**^*p <* 0.01; ^***^*p <* 0.001;

a: Effect of knowledge on sleep quality; b: Effect of sleep quality on depressive symptoms;

Model 1: Single factor analysis;

Model 2: Adjusted for age, grade, registered residence, any siblings, parents' education level, self-reported family economy, smoking, drinking;

Model 3: Adjusted for age, gender, registered residence, any siblings, parents' education level, self-reported family economy, smoking, drinking;

Model 4: Adjusted for age, gender, grade, registered residence, any siblings, parents' education level, self-reported family economy, smoking, drinking;

Table 3A Mediating effect of sleep quality between attitude and depressive symptoms

| Variable | Model | a | b | Direct Effect | Boot CI | |  | Indirect Effect | Boot CI | |  | MediationRatio, % |
| --- | --- | --- | --- | --- | --- | --- | --- | --- | --- | --- | --- | --- |
|  |  |  |  | c’ | LLCI | ULCI |  | (a×b) | LLCI | ULCI |  | (a×b)/(a×b+c’) |
| Gender |  |  |  |  |  |  |  |  |  |  |  |  |
| Male | 1 | -0.0421 | 0.3751^**^ | -0.4776^**^ | -0.6023 | -0.3529 |  | -0.0158 | -0.0403 | 0.0058 |  | - |
|  | 2 | -0.0439 | 0.3549^***^ | -0.4744^***^ | -0.5989 | -0.3498 |  | -0.0156 | -0.0394 | 0.0045 |  | - |
| Female | 1 | -0.0591^***^ | 0.5512^**^ | -0.4094^***^ | -0.5016 | -0.3467 |  | -0.0365 | -0.0558 | -0.0181 |  | 7.38 |
|  | 2 | -0.0695^***^ | 0.5245^***^ | -0.4241^***^ | -0.5027 | -0.3476 |  | -0.0372 | -00572 | -0.0188 |  | 7.92 |
| Grade |  |  |  |  |  |  |  |  |  |  |  |  |
| Freshman | 1 | -0.0684^***^ | 0.4951^***^ | -0.4649^**^ | -0.5592 | -0.3706 |  | -0.0338 | -0.0559 | -0.0131 |  | 6.78 |
|  | 3 | -0.0726^***^ | 0.4901^***^ | -0.4584^**^ | -0.5534 | -0.3635 |  | -0.0356 | -0.0581 | -0.0153 |  | 7.21 |
| Sophomore | 1 | -0.0517^*^ | 0.4739^***^ | -0.4255^***^ | -0.5154 | -0.3356 |  | -0.0245 | -0.0468 | -0.0033 |  | 5.44 |
|  | 3 | -0.0485^*^ | 0.4723^***^ | -0.4209^***^ | -0.5111 | -0.3308 |  | -0.0229 | -0.0457 | -0.0027 |  | 5.16 |
| Total |  |  |  |  |  |  |  |  |  |  |  |  |
|  | 1 | -0.0522^***^ | 0.5032 | -0.4358^**^ | -0.5013 | -0.3703 |  | -0.0263 | -0.0428 | -0.0108 |  | 5.69 |
|  | 4 | -0.0620^***^ | 0.4820 | -0.4403^**^ | -0.5059 | -0.3746 |  | -0.0299 | -0.0452 | -0.0154 |  | 6.36 |

^*^*p <* 0.05; ***p <* 0.01; ^***^*p <* 0.001;

a: Effect of attitude on sleep quality; b: Effect of sleep quality on depressive symptoms;

Model 1: Single factor analysis;

Model 2: Adjusted for age, grade, registered residence, any siblings, parents' education level, self-reported family economy, smoking, drinking;

Model 3: Adjusted for age, gender, registered residence, any siblings, parents' education level, self-reported family economy, smoking, drinking;

Model 4: Adjusted for age, gender, grade, registered residence, any siblings, parents' education level, self-reported family economy, smoking, drinking;

Table 4A Mediating effect of sleep quality between practice and depressive symptoms

| Variable | Model | a | b | Direct Effect | Boot CI | |  | Indirect Effect | Boot CI | |  | Mediation Ratio, % |
| --- | --- | --- | --- | --- | --- | --- | --- | --- | --- | --- | --- | --- |
|  |  |  |  | c’ | LLCI | ULCI |  | (a×b) | LLCI | ULCI |  | (a×b)/(a×b+c’) |
| Gender |  |  |  |  |  |  |  |  |  |  |  |  |
| Male | 1 | -0.0731^*^ | 0.3696^**^ | -0.3910^***^ | -0.5233 | -0.2587 |  | -0.0270 | -0.0537 | -0.0045 |  | 6.46 |
|  | 2 | -0.0679^*^ | 0.3524^***^ | -0.3793^***^ | -0.5115 | -0.2472 |  | -0.0239 | -0.0512 | -0.0022 |  | 5.92 |
| Female | 1 | -0.1819^***^ | 0.5309^***^ | -0.3297^***^ | -0.4161 | -0.2434 |  | -0.0966 | -0.1217 | -0.0720 |  | 22.66 |
|  | 2 | -0.1776^***^ | 0.5097^***^ | -0.3252^***^ | -0.4117 | -0.2387 |  | -0.0905 | -0.1154 | -0.0675 |  | 21.77 |
| Grade |  |  |  |  |  |  |  |  |  |  |  |  |
| Freshman | 1 | -0.1475^***^ | 0.4815^***^ | -0.3772^***^ | -0.4798 | -0.2746 |  | -0.0710 | -0.0985 | -0.0461 |  | 15.84 |
|  | 3 | -0.1461^***^ | 0.4786^***^ | -0.3699^***^ | -0.4726 | -0.2672 |  | -0.0699 | -0.0981 | -0.0459 |  | 15.89 |
| Sophomore | 1 | -0.1453^***^ | 0.4600^***^ | -0.3135^***^ | -0.4136 | -0.2134 |  | -0.0668 | -0.0946 | -0.0425 |  | 17.57 |
|  | 3 | -0.1378^***^ | 0.4587^***^ | -0.3129^***^ | -0.4133 | -0.2126 |  | -0.0632 | -0.0903 | -0.0395 |  | 16.80 |
| Total |  |  |  |  |  |  |  |  |  |  |  |  |
|  | 1 | -0.1483^***^ | 0.4857^***^ | -0.3471^***^ | -0.4193 | -0.2750 |  | -0.0720 | -0.0919 | -0.0538 |  | 17.18 |
|  | 4 | -0.1430^***^ | 0.4696^***^ | -0.3441^***^ | -0.4162 | -0.2719 |  | -0.0672 | -0.0863 | -0.0507 |  | 16.34 |

^*^*p <* 0.05; ^**^*p <* 0.01; ^***^*p <* 0.001;

a: Effect of practice on sleep quality; b: Effect of sleep quality on depressive symptoms;

Model 1: Single factor analysis;

Model 2: Adjusted for age, grade, registered residence, any siblings, parents' education level, self-reported family economy, smoking, drinking;

Model 3: Adjusted for age, gender, registered residence, any siblings, parents' education level, self-reported family economy, smoking, drinking;

Model 4: Adjusted for age, gender, grade, registered residence, any siblings, parents' education level, self-reported family economy, smoking, drinking;
